# Case Report: Autism-like phenomenology in functional neurological disorder in the social-media era: over- and under-diagnosis of autism as a bidirectional clinical problem

**DOI:** 10.3389/fpsyt.2026.1952291

**Published:** 2026-09-24

**Authors:** Pavlina Danhofer, Martina Vyhnalová, Lucie Stroupkova, Patricia Všianská, Lenka Knedlíková, Senad Kolář, Hana Ošlejšková

**Affiliations:** Department of Paediatric Neurology, University Hospital Brno and Faculty of Medicine of Masaryk University, Brno, Czechia

**Keywords:** alexithymia, ASD, autism, functional neurological disorder (FND), social media

## Abstract

Social media increasingly influence how adolescents and young adults understand and communicate neuropsychiatric symptoms, including symptoms attributed to autism spectrum disorder (ASD). This creates a particular diagnostic challenge in functional neurological disorder (FND), a condition in which neurological symptoms are genuine and disabling but are understood as arising from altered nervous-system functioning rather than structural neurological disease. Recent literature indicates that FND and ASD co-occur more often than would be expected by chance, although estimates vary substantially across studies and clinical populations. At the same time, autistic-like features such as sensory hypersensitivity, social difficulties, alexithymia, and altered interoceptive processing may occur across diagnostic boundaries and may therefore complicate differential diagnosis. The present case concerns a 17-year-old female patient with complex FND and prominent autistic-like phenomenology who attributed her difficulties to ASD largely through social-media exposure, while standardized assessment and retrospective developmental history did not support an ASD diagnosis. The case therefore provides an opportunity to examine two related but distinct questions: how genuine ASD may co-occur with FND, and how autistic-like experiences may arise in patients with FND without a developmental basis for ASD. We further consider whether, in a subset of patients, such phenomenology could represent a functionally mediated cognitive-behavioural construct shaped by predictive processing, interoception, alexithymia, and exposure to diagnostic narratives.

## 1 Introduction

Before considering the co-occurrence of these two conditions, brief working definitions are useful. Autism spectrum disorder (ASD) is a neurodevelopmental condition, present from early childhood, characterised by persistent differences in social communication and social interaction together with restricted, repetitive patterns of behaviour, interests, or activities, and frequently accompanied by atypical sensory reactivity; diagnosis rests on a developmental history consistent with these features from early life, supported where appropriate by standardised observational and historical instruments such as the ADOS-2 and ADI-R. Functional neurological disorder (FND) is a neurological condition in which motor, sensory, cognitive, or other neurological symptoms are genuine and disabling, are not explained by structural neurological or other medical disease, and are understood to arise from an alteration in nervous-system functioning that is, in principle, potentially reversible; diagnosis relies on positive clinical signs of internal inconsistency rather than on the exclusion of pathology alone. Although both are neurological/neuropsychiatric conditions with overlapping phenomenology, ASD is a developmentally anchored, lifelong condition, whereas FND is defined by its symptom mechanism and may fluctuate or resolve with treatment - a distinction that underlies much of the diagnostic complexity discussed below.

### 1.1 Functional neurological disorder, autism spectrum disorder, and the diagnostic role of social media

Functional neurological disorder (FND) and autism spectrum disorder (ASD) are clinically distinct conditions, but accumulating evidence suggests a meaningful degree of co-occurrence and phenotypic overlap. Recent systematic reviews and meta-analyses estimate that approximately 10% of patients with FND may have a co-occurring ASD diagnosis, although the estimates vary markedly according to age, FND subtype, clinical setting, and diagnostic methodology (1–6). Earlier studies likewise identified substantial overlap between functional neurological symptoms and autistic features, including sensory sensitivity, difficulties with emotion regulation, and altered interoception (1, 6). These findings make the differential diagnosis of ASD particularly relevant in FND services, while also highlighting the danger of interpreting overlapping traits as evidence of one disorder causing the other.

Against this background, social media represent an additional diagnostic influence rather than the primary explanation for the FND-ASD association. Social media platforms have become major sources of information about mental health and neurodevelopmental differences, particularly for adolescents and young adults. Repeated content analyses of autism-related posts show that a substantial proportion of available material is misleading, overly generalising, or clinically unsupported (7, 8). A systematic review of more than 5,000 posts from TikTok, YouTube, Facebook, Instagram, and X reported that approximately 40–41% of autism-related videos contained inaccurate or clinically unsupported information, while the corresponding proportion for ADHD-related videos exceeded 50% (1). A typical feature of inaccurate content is the presentation of transdiagnostic symptoms as specific to a single diagnosis, together with the description of common personality traits or stress responses as diagnostic criteria (2).

At the same time, not all autism-related social-media content is misleading or clinically unhelpful. Adults diagnosed with autism later in life frequently share subjective experiences under hashtags such as #actuallyautistic or #latediagnosedautistic. Such content may be perceived as authentic because it describes internal experiences that are less visible during a brief clinical assessment (9). For some individuals - particularly women and other people who have developed extensive compensatory strategies - this material may provide language for longstanding difficulties and may facilitate appropriate assessment rather than generate an incorrect diagnosis (10–14). Thus, social media may operate in opposite directions: they may contribute to premature self-attribution of ASD in some patients, while helping previously unrecognised autistic individuals identify a clinically relevant developmental history in others.

This distinction is particularly important in patients with FND because sensory hypersensitivity, fatigue, social exhaustion, alexithymia, dissociation, and difficulties interpreting bodily or emotional states may be present without being specific to ASD. The clinical task is therefore not to determine whether social media ‘caused’ an autism belief, but to establish whether the patient’s current autistic-like phenomenology is supported by a developmental history and standardized assessment, whether it is better explained by another psychiatric or functional process, or whether genuine ASD and FND coexist. (**Figure 1**). The present case is situated within this diagnostic tension: the patient presented with a persistent ASD self-narrative in the context of complex FND, but the available developmental history and standardized assessment did not support ASD.

**Figure 1:**
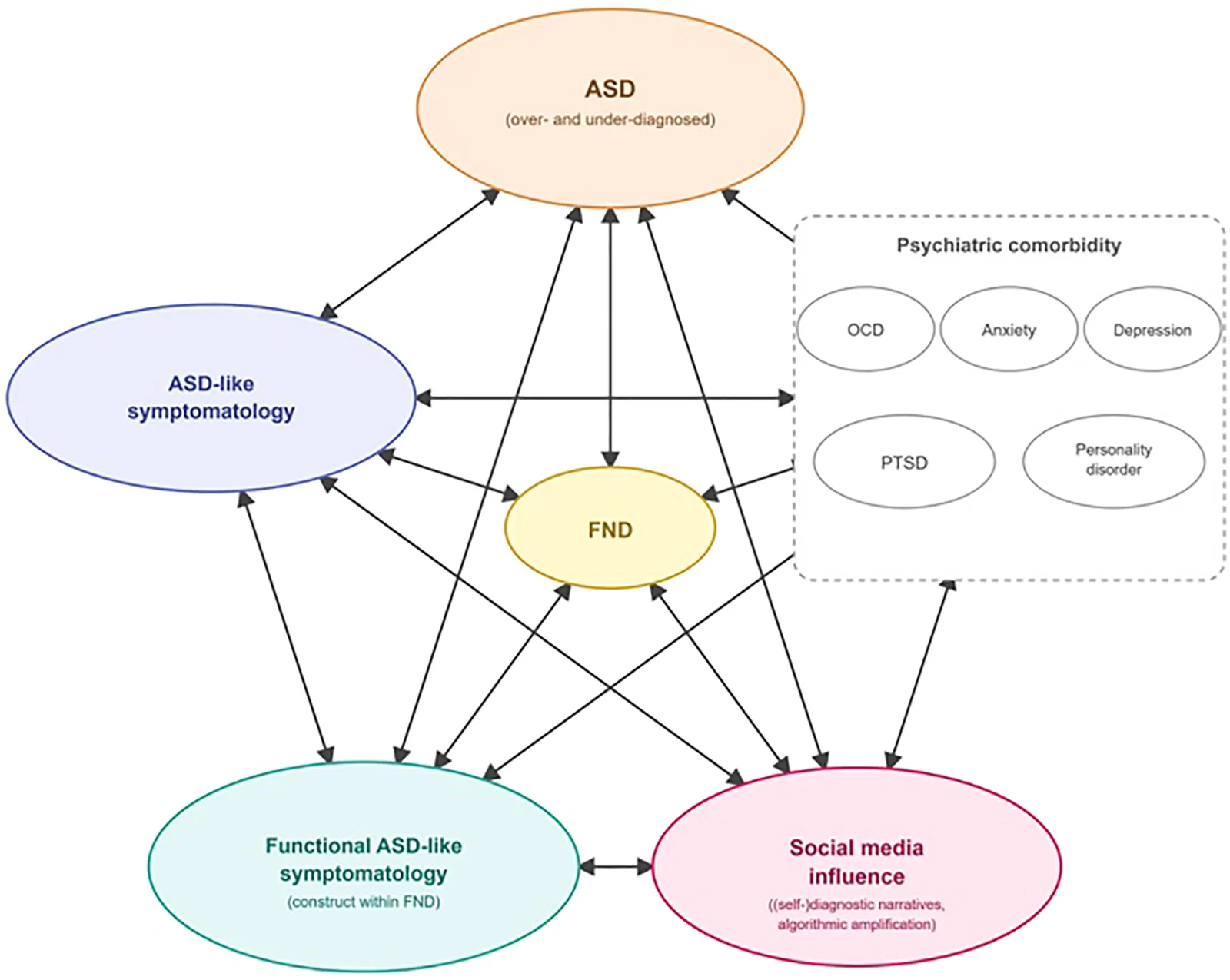
Conceptual model - ASD-like phenomenology, psychiatric comorbidity, FND, and social media influence.

### 1.2 Interoception, alexithymia, and the search for an explanatory framework

The overlap between FND and autistic-like phenomenology can be considered within a broader transdiagnostic framework involving interoception, alexithymia, emotion regulation, and predictive processing. These mechanisms are relevant across FND, obsessive-compulsive disorder (OCD), anxiety disorders, depressive disorder, personality disorders, and post-traumatic stress disorder (PTSD) (15–20). Importantly, their presence does not establish ASD, because the same mechanisms may contribute to similar subjective experiences across multiple diagnostic categories (**Figure 1**). Recent work on FND-ASD overlap likewise emphasizes common features involving sensory processing, interoception, and emotion regulation (16, 17).

According to the theory of constructed emotion, emotions are the result of active inference, whereby the brain interprets interoceptive signals through learned ‘emotion concepts’. When an individual lacks sufficiently differentiated emotion concepts - as may occur with alexithymia - ambiguous bodily signals such as rapid heartbeat, chest pressure, muscle tension, or fatigue may be more readily interpreted as symptoms of illness or dysfunction than as emotional states (17, 19). This mechanism is relevant to the present case because it provides a possible transdiagnostic explanation for why bodily and emotional experiences may be difficult to conceptualize without implying that they are intrinsically autistic.

For a patient who has long been unable to name their inner state, but who nonetheless experiences fatigue, sensory hypersensitivity, social exhaustion, a need for routine, and difficulties in social relationships, an autism narrative can provide a concrete and comparatively destigmatised explanatory framework. Adopting such a narrative may temporarily bring relief, comprehensibility, and a sense of belonging to a community that validates the person’s experience. In patients with emotionally unstable personality disorder, an autistic identity may additionally interact with a disrupted sense of identity; autism may be represented online as a neutral or positive neurodevelopmental difference, whereas personality disorder may be experienced as more stigmatizing (17). These mechanisms may help explain why an autistic self-narrative can persist despite negative standardized assessment, but they should remain hypotheses rather than conclusions inferred from a single case.

In the present patient, the persistence of the ASD self-narrative despite a negative ADOS-2 and an unremarkable retrospective developmental history is therefore clinically relevant, but it cannot by itself establish a functional autistic syndrome. Rather, it illustrates the need to distinguish the subjective reality of autistic-like experiences from the developmental criteria required for an ASD diagnosis.

### 1.3 The other side of the diagnostic spectrum: under-recognised autism and masking

The diagnostic problem is bidirectional. Some patients may attribute transdiagnostic difficulties to ASD without sufficient developmental evidence, whereas others - particularly girls and women - may have genuine ASD that remains unrecognised because of masking and compensatory social strategies. Standardized instruments such as ADOS-2 and ADI-R were developed and validated in populations that may not fully capture all presentations of autism, including subtler female phenotypes characterized by learned social scripts, imitation of peers, and internalised distress (10–12).

Over the course of their lives, some autistic individuals develop sophisticated compensatory strategies, often described as camouflaging. They may therefore appear socially competent during brief clinical contact while experiencing substantial anxiety, exhaustion, and a persistent sense of being different. Camouflaging has been associated with psychological exhaustion, burnout, and delayed access to appropriate care.

These patients may consequently accumulate other psychiatric diagnoses - including anxiety disorders, depression, OCD, PTSD, and personality disorders - before ASD is considered (13, 14). (**Figure 1**). Available studies suggest that diagnostic errors and delays are particularly relevant in autistic women, although the precise frequency of misdiagnosis varies across samples and should not be generalized to all women with ASD (10–14).

A large survey of adults with ASD found that approximately a quarter of respondents reported at least one previous psychiatric diagnosis they considered incorrect, with women reporting perceived misdiagnosis more often than men, particularly involving personality, anxiety, and affective disorders (14). These findings reinforce the need for developmental history and clinical formulation rather than reliance on either a patient’s self-identification or a single standardized instrument.

For this group, social media may paradoxically play a helpful role: content created by late-diagnosed autistic individuals can provide language for previously unexplained experiences and prompt appropriate professional assessment. Therefore, an autistic self-identification acquired online should neither be automatically accepted nor dismissed. In patients with FND, the appropriate response is a structured assessment that considers developmental history, current symptom profile, standardized ASD measures, functional neurological symptoms, and alternative explanations.

The clinical difficulty is that both groups may report an apparently similar subjective picture - sensory hypersensitivity, social difficulties, alexithymia, somatic symptoms, and an autistic self-narrative. Distinguishing developmental ASD from functionally mediated or transdiagnostic autistic-like phenomenology therefore requires careful assessment rather than symptom counting alone. The epidemiological evidence for FND-ASD co-occurrence further underscores why this distinction matters: the presence of FND should not be used to exclude ASD, just as autistic-like features should not be assumed to establish ASD (1–4).

### 1.4 Aim of the study

The aim of this paper is to present a case of complex FND with psychiatric comorbidities and prominent autistic-like symptomatology in a patient whose ASD self-identification was not supported by standardized assessment or retrospective developmental history. The case is discussed within the broader literature on FND-ASD co-occurrence, with particular attention to the diagnostic implications of social-media-derived narratives. We propose, cautiously, that in a subset of patients with FND, autistic-like phenomenology may be functionally mediated and may interact with predictive processing, interoception, alexithymia, emotion regulation, and identity-related processes. This hypothesis is intended to complement - not replace - the established possibility of genuine ASD-FND comorbidity.

## 2 Case presentation

A 17-year-old female patient was referred for neuropsychological assessment in April 2023. Her functional neurological symptoms had developed in 2021, following her return to school after the COVID-19 pandemic. She required full-time wheelchair use because of functional lower-limb weakness, met clinical criteria for functional seizures and functional tic-like behaviours, and reported prominent sensory hypersensitivity to sound and light, alongside fatigue, insomnia, fibromyalgia, and joint hypermobility. She had independently concluded - primarily through social media engagement - that she had ASD, and presented requesting formal assessment. She described social uncertainty, difficulty forming relationships, problems reading social norms, limited use of facial expression and intonation, habitual social masking, and a pervasive sense of being “different.” Marked distrust of medical professionals was evident throughout the assessment.

Retrospective developmental history obtained from her parents was unremarkable for ASD-specific features: language and social development had proceeded age-appropriately, play was typical, and no stereotyped interests, ritualistic behaviours, or early sensory difficulties were reported. Social isolation had emerged in parallel with FND onset. ADOS-2 administration did not yield scores meeting the diagnostic threshold for ASD. Projective assessment revealed uncertainty in interpreting emotions alongside preserved social understanding and mentalising capacity. The overall assessment concluded a disharmonious personality development without fulfilment of ASD diagnostic criteria. As part of the comprehensive assessment, no depressive symptoms were identified in the patient’s clinical presentation, history, or psychological test results. The patient reported increased anxiety in the context of social interactions; no other manifestations of anxiety were identified. Throughout this process, the patient maintained a firm conviction in her self-diagnosis, returning to the topic repeatedly despite negative findings - a pattern consistent with the identity-consolidation mechanism described above.

Following interdisciplinary rehabilitation, independent ambulation was achieved. From early 2024, however, progressive deterioration occurred: weakness extended to a quadri-distributed pattern, seizure and tic frequency increased, and functional urinary incontinence emerged in 2025, with generalised pain becoming the dominant complaint. The patient is currently managed under a primary diagnosis of emotionally unstable personality disorder with complex FND comorbidity. This trajectory illustrates how an unresolved autistic-like phenomenology-FND presentation - including a persistent ASD conviction and the identity functions it serves - can interact with underlying personality pathology to maintain and amplify functional symptoms over time.

## 3 Discussion

### 3.1 Epidemiological evidence for the co-occurrence of FND and ASD

The co-occurrence of FND and ASD is increasingly supported by epidemiological and clinical studies, although the magnitude of the association remains uncertain. Gonzales-Herrero et al. reported that among 344 patients with FND, 8% had a confirmed ASD diagnosis and a further 69% showed clinically significant autistic traits (1). More recent systematic reviews and meta-analyses provide a broader estimate of the frequency of co-occurrence. Vickers et al. found evidence that ASD is more common in FND populations than in the general population and reported approximately 10% comorbidity among children presenting with functional seizures, while also identifying evidence of increased ASD rates in other FND presentations (4). Tamilson et al. synthesized 24 studies including 11,324 participants and estimated the proportion of ASD among people with FND at approximately 10% (95% CI 7–15%), with substantial heterogeneity between studies and clinical subgroups (3). The same review estimated ASD in approximately 9% of adults and 10% of children with FND, while higher proportions were observed in functional tic-like behaviours and lower proportions in functional seizures (3).

A large retrospective cohort analysis identified substantial numbers of patients with co-occurring ASD and FND and suggested that ASD was frequently diagnosed only after FND (3, 4). Such findings may indicate that contact with specialist neurological services creates opportunities to recognize previously unrecognised neurodevelopmental conditions. However, they may also partly reflect differences in ascertainment, referral patterns, and diagnostic practices. Accordingly, the epidemiological literature supports a clinically important association between FND and ASD, but does not by itself establish shared aetiology or a direct causal relationship.

Taken together, these data support the hypothesis that FND and ASD may be associated more often than would be expected from population prevalence alone, while the mechanisms underlying this association remain uncertain (1–4). The present case does not provide evidence of ASD-FND comorbidity; rather, it illustrates the complementary diagnostic problem of autistic-like phenomenology in a patient with FND who did not meet ASD criteria. The distinction between these two clinical situations is central to the interpretation of the case and illustrated by **Table 1**.

**Table 1:** Differential diagnostic comparison of ASD and autistic-like phenomenology across twelve clinically relevant dimensions.

Dimension	ASD	Autistic-like phenomenology
Onset and course	Neurodevelopmental; symptoms present from early childhood, stable and lifelong.	No neurodevelopmental onset; features emerge with or after FND, variable course.
Social difficulties	Qualitative impairment in reciprocal social interaction; consistent across contexts.	Increased cognitive effort and discomfort in social situations; variable, partly driven by fatigue and emotional dysregulation.
Sensory processing	Atypically established in development; present across contexts and modalities.	Hypersensitivity concurrent with FND onset; fluctuates with symptom severity and attentional state.
Repetitive/restricted behaviour	Core defining feature with regulatory and self-stimulatory function.	Behavioural rigidity secondary to anxiety, fatigue, and intolerance of uncertainty; not a primary feature.
Neurobiological substrate	Stable, trait-level alterations in cortical connectivity, established pre- or perinatally.	State-dependent functional network dysregulation; potentially reversible with treatment.
Alexithymia	Present in ~50% of ASD individuals; co-occurring trait, not diagnostic criterion.	Frequently elevated; proposed as a core mediating mechanism
Interoceptive accuracy	Heterogeneous findings; not consistently reduced.	Typically reduced; contributes to symptom misattribution and susceptibility to external interpretive frames.
Identity and self-narrative	ASD diagnosis typically integrates with existing self-understanding.	ASD self-narrative frequently adopted via social media; may serve identity-stabilising function in personality pathology.
ADOS-2 outcome	Meets diagnostic threshold.	Does not meet diagnostic threshold; symptom endorsement without meeting observational criteria.
Temporal variability	Core features stable over time and across situations.	Features variable; modulated by fatigue, emotional state, context, and attention – characteristic of FND.
Response to FND treatment	Not modified by FND-specific interventions.	Social-cognitive and sensory features may improve alongside functional symptom reduction.
Developmental history	Retrospective history supports early atypical development.	Developmental history unremarkable for ASD-specific features; social difficulties arise with FND.

### 3.2 Shared pathophysiology: predictive processing, interoception, and connectivity

From the perspective of computational neuroscience, FND is often conceptualised as a disorder of predictive processing: symptoms arise when high-level, body-related expectations (priors) are assigned disproportionately high precision weighting within the processing hierarchy at the expense of actual sensory data, generating persistent prediction-error signals that give rise to genuine neurological symptoms (16, 17).

In ASD, the situation appears, at first glance, to be the opposite: the HIPPEA framework (High, Inflexible Precision of Prediction Errors in Autism) instead describes an excessive and inflexible weighting assigned to prediction errors at lower levels of processing, i.e., difficulty filtering sensory “noise.” Crucially, however, both configurations may lead to the same compensatory response: if the system cannot adequately dampen inaccurate but highly salient sensory signals, it may generate, at higher levels of the hierarchy, a strongly weighted, maladaptive “bodily” prior intended to impose coherence on chaotic input - a mechanism considered central to the generation of functional neurological symptoms (15, 17). In other words, the autistic brain, owing to difficulty filtering sensory noise, may be particularly vulnerable to the emergence of the maladaptive, symptom-generating priors described in models of FND - which would explain why the risk of developing FND is increased in autistic individuals, without requiring the assumption of a single shared causal pathway (17).

A second dimension of the shared mechanism is interoception and alexithymia. Alexithymia is present in 35–75% of patients with FND and in 40–60% of autistic individuals; in both populations, a so-called interoception trait prediction error (ITPE) has been described - a mismatch between objectively measured interoceptive accuracy (typically reduced) and subjective confidence in one’s ability to interpret bodily signals (typically increased) (21–23).

In patients with functional seizures, higher ITPE strongly predicts the degree of dissociation and correlates with reduced white-matter integrity originating from the bilateral insula and the temporoparietal junction - regions critical for the integration of multisensory information and for the sense of body ownership. These regions, together with the anterior cingulate cortex, are also repeatedly described as sites of atypical connectivity in ASD (20, 21).

Taken together, the literature provides converging but still incomplete evidence for overlap between FND and ASD in domains including interoceptive processing, alexithymia, sensory processing, and emotion regulation. This overlap should be interpreted as a plausible area of shared vulnerability rather than proof of a common pathophysiological pathway. ASD represents a neurodevelopmental condition with developmentally anchored features, whereas FND is a functional neurological disorder whose symptoms may fluctuate with context and can be potentially reversible. The distinction is important when interpreting autistic-like features in an individual patient.

### 3.3 Functional cognitive disorder and functional tic-like behaviour as precedents

In recent years, the spectrum of FND has expanded to include entities that do not primarily present with motor or sensory symptoms but with complex cognitive and behavioural pictures. Functional cognitive disorder describes subjectively pronounced difficulties with attention, memory, and executive function that cannot be explained by structural brain pathology and, like other FND symptoms, are modulated by attention and context.

This logic is illustrated even more clearly by the phenomenon of rapid-onset functional tic-like behaviour, popularised in connection with the COVID-19 pandemic and so-called “TikTok tics”: in adolescents, predominantly girls, a complex picture resembling Tourette syndrome developed over a short period of time, in direct temporal association with the consumption of videos depicting tics on social media, without any underlying organic tic disorder (24–26).

This phenomenon is today - although still debated - described in the literature as a consequence of observational learning and predictive processing: repeated exposure to a behavioural model on social media generates a highly weighted prior for a particular motor pattern, which is subsequently genuinely, involuntarily enacted. It thus represents a fully-fledged functional neurological symptom whose content (the specific form of the tic) was nonetheless “supplied” externally, via social media (24).

Importantly, rapid-onset functional tic-like behaviour provides a useful precedent for considering how social-media exposure can influence symptom content without implying that all FND symptoms are socially learned. Repeated exposure to behavioural models may contribute to the formation of highly weighted expectations for particular motor patterns in susceptible individuals, which may subsequently be experienced as genuine and involuntary. This does not demonstrate that social media generate autistic phenomenology in the same way. Rather, it provides a conceptual example of how socially available symptom narratives can interact with pre-existing vulnerabilities in functional disorders.

### 3.4 Is autistic-like symptomatology in FND a complex construct of FND itself?

On the basis of the epidemiological and mechanistic literature, we can formulate a more cautious hypothesis: in a subset of patients with FND, autistic-appearing symptomatology - such as social difficulties, sensory hypersensitivity, need for routine, or alexithymia - may not be fully explained by comorbid anxiety, somatisation, or personality pathology. (**Figure 1**) In some cases, these experiences may become organized into an autistic narrative through processes involving attention, predictive processing, interoception, emotion regulation, and social learning. However, the present case cannot establish that such phenomenology constitutes a distinct subtype of FND; this remains a hypothesis requiring prospective investigation.

In the present case, the autistic ‘content’ of the patient’s experience was clinically real and distressing, but the available developmental history and ADOS-2 findings did not support ASD. Social-media exposure may have contributed to the organization and persistence of the patient’s interpretation of these experiences, but causality cannot be inferred. The analogy with functional tic-like behaviour should therefore be regarded as conceptual rather than evidentiary: unlike a functional tic, autistic-like social and sensory characteristics are multidimensional and may arise from numerous developmental, psychiatric, personality, and functional processes.

This hypothesis does not contradict the established observation that autistic individuals may be more likely to experience FND or functional neurological symptoms, potentially in association with sensory sensitivity, alexithymia, anxiety, somatisation, or cumulative psychosocial burden. Genuine ASD and FND may coexist in the same patient, and such comorbidity should not be overlooked simply because autistic traits can also occur in patients without ASD.

The clinically relevant question is therefore not whether autistic-like phenomenology is ‘real’ versus ‘fake’, but what developmental and functional mechanisms best account for it in a particular patient. In patients with FND and autistic-like symptoms, clinicians should distinguish at least three possibilities: (1) genuine ASD with co-occurring FND; (2) autistic-like symptoms arising from transdiagnostic psychiatric or personality-related processes; and (3) a functionally mediated pattern of autistic-like phenomenology that may be shaped by attention, predictive processing, interoception, and socially available diagnostic narratives. Current evidence is insufficient to establish the third category as a formal diagnosis.

If this hypothesis is eventually supported, functionally mediated autistic phenomenology would represent neither conventional ASD overdiagnosis nor simple under-recognition, but a clinically meaningful pattern requiring a formulation similar to other functional syndromes. At present, however, the evidence supports using this concept as a research hypothesis rather than a diagnostic category. For clinical practice, the priority remains rigorous differential diagnosis, validation of the patient’s subjective experience, and treatment directed at established functional and transdiagnostic mechanisms rather than confirmation or confrontational refutation of an unsupported diagnostic label.

### 3.5 Clinical implications

Five clinically relevant conclusions follow. First, the epidemiological literature indicates that ASD and FND can co-occur and that ASD assessment is therefore appropriate when autistic features are clinically prominent. Second, in patients presenting with FND and autistic-appearing symptomatology, standardized ASD assessment (e.g., ADOS-2) should be combined with a careful collateral developmental history; neither a positive self-identification nor a single negative instrument should be considered sufficient in isolation. Third, in women and other individuals with a high degree of masking, clinicians should actively enquire about developmental social communication differences, compensatory strategies, and histories of diagnostic delay or perceived misdiagnosis. Fourth, social-media exposure should be explored neutrally: clinicians should ask what content the patient encountered, which experiences they recognized in themselves, and whether the online narrative preceded or followed the onset of symptoms. In the present case, this is directly relevant because the patient’s ASD formulation emerged in the context of social-media engagement, while the developmental history and ADOS-2 did not support ASD. While social media are the focus of this discussion, comparable interpretive influence may also arise from family members, peers, or prior clinical contact; a systematic exploration of these sources was beyond the scope of the present case. Fifth, in patients in whom standardized assessment does not confirm ASD, but the autistic self-narrative persists and is entangled with complex FND symptomatology, it may be preferable to offer a transdiagnostic framework focused on interoception, alexithymia, emotion regulation, and the predictability of one’s own bodily reactions-mechanisms shared by FND, autistic-like phenomenology, and a range of anxiety, depressive, and personality-related constellations described in the Introduction.

## 4 Conclusion

Social media can play an active role in shaping diagnostic narratives among patients with FND and related psychiatric conditions, but this role should be understood within the broader context of the clinically important co-occurrence of FND and ASD. Current epidemiological evidence suggests that ASD is more frequent among people with FND than would be expected from population prevalence alone. This association warrants routine clinical awareness of possible ASD, while the substantial heterogeneity of available studies requires caution in interpreting prevalence estimates or inferring shared causation.

The present case illustrates a different but complementary diagnostic problem: a patient with complex FND and prominent autistic-like phenomenology whose ASD self-identification was strongly influenced by social media but was not supported by standardized assessment or retrospective developmental history. The case therefore should not be interpreted as evidence that social media cause autistic-like symptoms, nor as evidence against ASD in patients with FND. Instead, it highlights how an autistic explanatory narrative may become persistent and clinically consequential in a patient whose difficulties are better conceptualized within FND and associated psychiatric processes. The proposed hypothesis—that a subset of patients with FND may develop functionally mediated autistic-like phenomenology—remains speculative and requires prospective validation using standardized ASD assessment, developmental histories, measures of interoception and alexithymia, and longitudinal assessment of symptom development in relation to social-media exposure.

Clinically, these findings support a careful, non-confrontational, and developmentally informed differential diagnosis. The goal is to avoid both overdiagnosis and under-recognition of ASD while validating the patient’s actual symptoms and disability. In patients with confirmed ASD and FND, treatment should address both conditions; in patients without developmental evidence for ASD, treatment may instead focus on established functional mechanisms and transdiagnostic targets such as interoception, alexithymia, emotion regulation, and attentional processes (27). The case thus supports a formulation-based approach in which the ASD-FND association is taken seriously without assuming that autistic-like phenomenology necessarily represents ASD.

## Data Availability

The raw data supporting the conclusions of this article will be made available by the authors, without undue reservation.
